# Permittivity Measurements for Cypress and Rockrose Biomass Versus Temperature, Density, and Moisture Content

**DOI:** 10.3390/s20174684

**Published:** 2020-08-19

**Authors:** Rafael Pérez-Campos, José Fayos-Fernández, Antonio José Lozano-Guerrero, Antonio Martínez-González, Juan Monzó-Cabrera, Irene Mediavilla, David Peña-Carro, Luis Saúl Esteban-Pascual

**Affiliations:** 1 Departamento de Tecnologías de la Información y las Comunicaciones, Universidad Politécnica de Cartagena, 30202 Cartagena, Spain; rafael.perez@upct.es (R.P.-C.); jose.fayos@upct.es (J.F.-F.); antonio.lozano@upct.es (A.J.L.-G.); toni.martinez@upct.es (A.M.-G.); 2 Centro de Desarrollo de Energías Renovables, CEDER-CIEMAT, 42290 Lubia, Spain; irene.mediavilla@ciemat.es (I.M.); David.Pena@ciemat.es (D.P.-C.); luis.esteban@ciemat.es (L.S.E.-P.)

**Keywords:** cypress, rockrose, biomass, permittivity, resonant technique, dielectric constant, loss factor

## Abstract

Permittivity of materials is of utmost importance for microwave applicators’ design and to predict high-frequency dielectric heating of materials. In the case of aromatic plant biomass, however, there are few data that help researchers design microwave applicators for microwave-assisted extraction. In this work, the permittivity of cypress and rockrose biomass samples were measured versus temperature, density, and moisture content. A resonant technique based on a coaxial bi-reentrant microwave cavity was employed to obtain the complex permittivity of biomass samples as a function of those magnitudes around the 2.45 GHz ISM frequency. The obtained measurements show that large variations for permittivity values can be found with moisture content and density changes for both cypress and rockrose biomass. Temperature also has effects in a lesser degree, although it has an important influence on the cypress biomass loss factor. Polynomial expressions fitting the experimental data were provided in order to facilitate the estimation of intermediate values, which were not explicitly arranged in this work. As a general trend, the permittivity of cypress and rockrose biomass increases with increasing values of moisture content and density, whereas the biomass loss factor increases when temperature rises.

## 1. Introduction

Chemical products contained in plants have been used in traditional medicine, pharmaceutic and perfumery products, and as a source of antioxidant treatments. Essential oils (EOs) contained in aromatic plants may, therefore, be valuable products provided that they are economically extracted.

In Spain, the biomass of common plants, such as cypress or rockrose, may be a good source of EO extraction since they are commonly available after forest enhancement treatments, such as pruning and clearing and sustainable logging exploitation, increasing the added value of silviculture. EO and methanol extracted from Mediterranean cypress (*Cupressus sempervirens* L.) possess antimicrobial and antibiofilm properties, and therefore can be used as natural preservative ingredients in food and/or pharmaceuticals [1]. Rockrose (*Cistus ladanifer*) is also widely spread in the Mediterranean area, and may be a good source of natural antioxidants since extracts from this plant show methanolic, phenolic, and flavonoid contents as described in [2]

Microwave-assisted extraction (MAE) of EOs and other valuable chemical compounds from aromatic herbs, and its variants, has been actively investigated during the last decades since it promises substantial cost and time savings and provides greater yields than conventional procedures. In [3,4], for instance, MAE was applied to basil, garden mint, thyme, and other species, such as ajowan, cumin, and star anise, without using any solvent to collect those essential oils.

MAE of phenolic substances from aromatic plants was also studied in [5] by using different solvents. The results in this work show that time and solvent use were reduced and the extraction of phenolic compounds was increased versus conventional techniques.

The modelling and optimization of energy consumption for conventional and different MAE extraction procedures in many aromatic plants were proposed and tested in [6]. In this work, the authors showed that MAE consumed less energy than conventional treatments in almost all studied cases and that a compromise between the employed energy and extraction efficiency exists.

Microwave-assisted hydro-distillation (MAHD) was used for the extraction of essential oil from *O. vulgare* L. ssp. Hirtum [7]. MAHD proved to be a technique with many advantages compared to conventional hydro-distillation, such as a shorter extraction time, better yields of essential oil, higher content of oxygenated compounds, and lower electrical consumption figures.

MAE processes’ efficiency greatly depends on the relative permittivity of aromatic plants and the microwave applicator design, and therefore the characterization of the dielectric constant and loss factor as a function of the temperature, density, and moisture content must be carried out in order to properly design industrial microwave applicators capable of obtaining high energy efficiency values and extraction yields.

Dielectric properties’ determination for every kind of material has been a widely researched issue for many years in the area of microwave applications. When materials are to be treated with microwaves for different purposes, such as drying, online moisture measurements, disinfestation, remote sensing, and others, thorough knowledge of the material’s dielectric properties becomes extremely important.

These measurements are commonly carried out as a function of the moisture content and microwave frequency. Additionally, in the area of biological and organic materials, an extensive activity, for determining dielectric properties, has been published in the literature and, specifically, many studies are about plants [8,9,10,11,12,13,14,15,16,17,18,19].

In [8,9], an application of microwave measurement techniques for determining the permittivity was proposed in the field of agricultural products. A similar technique is presented in [10,11] for fresh leaves from different plant species. In [12,13], dielectric models that only require measurement of the moisture content (MC) were investigated for alfalfa within different frequency ranges. In [14], the dielectric response of corn leaves to water stress was analyzed for radar applications. Other plants like wheat [15], two types of conifers [16], apples [17], and holly leaves in frozen environments [18] have also been investigated. Other authors dealt with the issue of seasonal differences in the dielectric properties of dwarf woody tundra vegetation [19].

For other specific plants, there are not yet many studies published about their dielectric properties. It is, in general, the case of aromatic plants like rockrose and cypress. An application to low-moisture rosemary can be found in [20]. Nevertheless, the extraction of EO from aromatic herbs is an industrial process that can be conducted with the aid of microwave energy as an alternative to conventional hydro-distillation [21], and permittivity measurements are necessary for equipment design.

The frequency range is, generally, one of the main aspects regarding dielectric measurements of materials. The different studies mentioned range from an extremely low frequency (<1 kHz) [17], going by 1–1000 Hz [10], 1.4 GHz [19], 2.45 GHz [20], 3.5 GHz [14,15], 5.5–7.5 GHz [21], 7.5–8.5 GHz [15], 1–10 GHz [3], 300 MHz–11 GHz [17], 300 MHz–18 GHz [12,13], 0.5–40 GHz [18], and up to 100 GHz [11].

To the best knowledge of the authors, however, there are not any works that describe the dielectric properties of cypress or rockrose biomass as a function of the temperature, density, or moisture content. Therefore, in this work, we present permittivity measurements for the biomass of these aromatic plants so that it can be further used in order to properly design efficient microwave applicators.

There are many techniques to obtain the permittivity of any kind of material. Additionally, there is a wide extension of literature containing data with the electrical properties of materials at different frequencies. Available techniques can be divided into resonant and non-resonant [22]. Resonant methods can also be subdivided into resonant cavities, open resonators, and dielectric resonators [23]. Additionally, sensing electromagnetic structures can be implemented using different transmission lines. Coaxial, waveguide, and microstrip lines are some usual examples to implement the measuring circuit [24].

Some of these techniques use open configurations, where part of the energy is radiated into the ambient like in the free-space measurements, coaxial probes, or microstrip lines, while others show closed configurations, where the energy is confined in the measurement device like in a waveguide setup. Another classification can be made attending to the use of one port (reflection measurement) or two ports (transmission measurement) [24]. In most of the cases, the sample is placed in a holder and this requires adapting the sample, or destructing it, to exactly fit it into its dimensions. Consequently, techniques can also be divided into destructive or non-destructive.

The rationale behind selecting one measuring technique in between the number of existing ones lies in the working frequency, the expected electrical losses of the material under test (MUT), and its homogeneity, shape, and adaptability to the holder. If the study must be carried out by varying the temperature, the existing techniques must be adapted with this purpose and some of them can be more easily adapted than others.

The conditions of the measurement process prevented authors from using other techniques like Nicholson Ross Weir in a closed waveguide configuration or open coaxial probes with available commercial kits from the brands SPEAG or Keysight. These last ones were unsuitable due to the material’s shape and its heterogeneity.

The selected device for the measurements uses a resonant technique that provides results nearby the frequency of interest, 2.45 GHz, and the sample was placed in a Pyrex vial that fits into a cylindrical cavity. The sample can be previously heated to obtain its properties while the temperature decreases. Resonant techniques use the frequency shift to provide the dielectric constant of the sample and the quality factor Q to obtain the loss factor. Although this technique is usually preferred for low-loss materials, it has been adapted, including the de-embedding of the feeding network to be suitable for high-loss materials [23].

The obtained results show that, as a general trend, the permittivity of cypress and rockrose biomass increases with increasing values of the moisture content and density, whereas the biomass loss factor increases when the temperature rises.

## 2. Materials and Methods

### 2.1. Permittivity Measurement Equipment

The heterogeneous composition of the biomass used in this study (fine twigs with leaves) and its handling limit the techniques suitable for its permittivity measurement. For instance, the coaxial probe technique may produce important errors due to the air gaps present in the biomass composition. As temperature variation is also important to predict the behavior of the biomass complex permittivity during MAE procedures, waveguide techniques are complex to implement. Additionally, the impedance technique is limited in frequency and sample thickness. Therefore, resonant procedures seem to be the best ones to suit the conditions of heterogeneity, temperature, density, and moisture content during the permittivity measurement of cypress and rockrose biomass.

Thus, a Dielectric Kit for Vials (DKV) from ITACA institute [25] was used to perform all the relative complex permittivity measurements. This equipment can determine the dielectric constant and loss factor of a wide range of liquid, granular, or powdered materials around 2.45 GHz. Its measurement procedure is based on a resonant technique and, therefore, the real and imaginary part of the relative permittivity are estimated by measuring the changes of the resonant frequency and quality factor of the instrument with and without material. This standalone equipment implements a resonant coaxial bi-reentrant microwave cavity, where the material under test is placed inside a vial and the complex permittivity is obtained by using numerical methods based on mode-matching and circuit analysis. Although resonant techniques are usually preferred for low-loss materials, the technique employed by DKV includes the de-embedding of the feeding network to be suitable for high-loss materials [22].

The DKV operates in a frequency bandwidth ranging from 1.5 to 2.6 GHz. It can provide dielectric constant values lower than 100 and loss factor values ranging from 0.001 to 15. The accuracy for the measurements is around 1% for the dielectric constant and a minimum of 5% accuracy is ensured for the loss factor. Repeatability and linearity values provided by manufacturer are around 0.2%.

During the permittivity measurements of both cypress and rockrose biomass samples, the resonant frequencies of the equipment ranged from 2.464 to 2.561 GHz and from 2.390 to 2.562 GHz, respectively. It must be noticed that the selected device provides the permittivity result for just one frequency nearby 2.45 GHz and cannot provide a wide-band characterization, which would be more useful. However, since the purpose of the study was to obtain the permittivity of the material for microwave-heating purposes at the specified ISM frequency, its use seemed reasonable.

Obviously, this becomes a disadvantage if the characterization for other interesting ISM frequencies like 915 or 27 MHz is needed. In this case, another measuring alternative should be evaluated.

### 2.2. Biomass Sample Preparation

Two sorts of biomass were studied in this work: Mediterranean cypress and rockrose plants collected in the province of Soria (Spain). For each kind of plant, the samples were prepared by introducing a biomass of cypress or rockrose ranging from 1 to 1.8 g in 6-mL quasi-cylindric polypropylene test tubes (Deltalab Ref. 400400) with an internal diameter of 10.3 mm and a height of 96.9.

Biomass materials (twigs) were collected manually and were prepared by milling 10 kg in a hammermill with a screen pore size of 10 mm.

**Figure 1:**
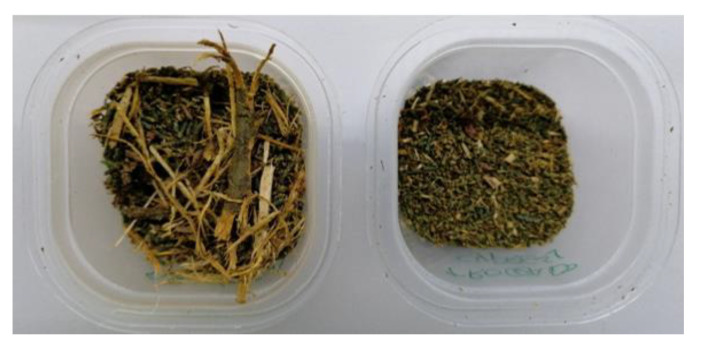
Cypress biomass before (**left**) and after (**right**) being chopped.

Each sample of cypress and rockrose biomass contained leaves, small limbs, stalks, etc. in a heterogeneous mixture, where the largest pieces had a length of up to 40 mm and the shortest pieces did not even reach 2 mm. In order to avoid the uncertainty generated by this heterogeneity and to reduce airgaps, the largest pieces were chopped in pieces with a maximum length of 5 mm as shown in Figure 1 and then introduced in the polypropylene tubes. This sample conditioning is a compromise between the biomass milling process carried out before the microwave-assisted hydro-distillation process and the granulate specifications of the DKV instrument.

For each biomass sample, three different permittivity measurements were carried out and the average value of the three measurements was used as the valid measurement. This procedure was done in order to reduce the uncertainty of the procedure.

Additionally, the permittivity measurements depending on the temperature, moisture content, and density were carried out by averaging the results of three different samples as explained in the next sections.

### 2.3. Measurement Procedure for Temperature Dependence of Biomass Complex Permittivity

**Figure 2:**
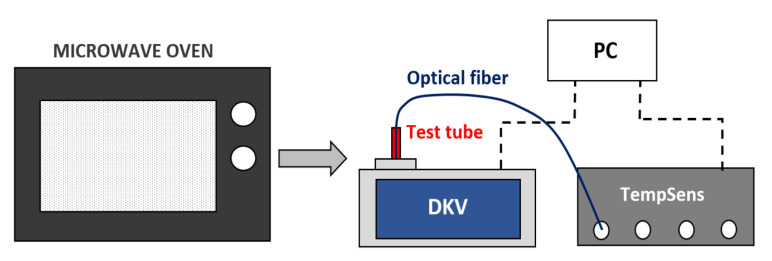
Setup used for measuring the permittivity complex dependence on temperature.

The procedure for measuring the permittivity of biomass samples as a function of temperature is similar to the one described in [26]. Figure 2 shows a scheme of the setup employed for measurements. The sample to be measured was introduced in a 150-mL glass of water and then was heated in a conventional microwave oven until the water boiled.

The polypropylene test tube was then rapidly dried in order to avoid any presence of little drops on its external surface that might ruin the measured dielectric properties and then it was introduced in the sample container of the DKV. An optical fiber was then introduced in the biomass sample in order to collect the temperature data. A Labview program was used to control the TempSens thermometer from OpSens and to collect both the biomass temperature and time data [27]. The TempSens temperature accuracy is equal to ±0.8 °C or better for temperatures higher than 45 °C and equal to ±0.3 °C or better for temperatures lower than 45 °C.

At the same time, the DKV saved the relative complex permittivity and the time data in a txt file. Both temperature and permittivity text files were processed in Matlab to assign the dielectric properties to the measured temperature data by comparing temperature and permittivity time vectors. It must be remarked that data from the three tests for every biomass sample were collected and averaged for each complex permittivity value.

The sample temperature was measured following Newton’s law of cooling and therefore more permittivity data were obtained for lower temperatures than for higher ones since the sampling rate for DKV cannot be modified and temperature decreases faster for higher temperatures. Therefore, in order to average the permittivity measurements with the similar temperature, we grouped the biomass permittivity data obtained for the 9 tests, corresponding to 3 samples and 3 tests per sample, within 1 °C ranges and averaged them in each temperature range.

For both cypress and rockrose biomass measurements, the first temperature measurement group was contained between 84.5 and 85.5 °C and the averaged obtained values were assigned to 85 °C, and the final temperature group was contained between 29.5 and 30.5 °C and the averaged values were assigned to 30 °C.

The moisture content and the density of all samples were kept constant during these measurements. For the cypress biomass permittivity measurements depending on temperature, the dry-basis moisture content for samples was around 100% and the density was close to 0.34 g/cm^3^. In the case of the rockrose permittivity measurements depending on temperature, those values were 100% and 0.3 g/cm^3^ for the moisture content (dry basis) and density, respectively.

### 2.4. Measurement Procedure for Biomass Permittivity Variation Versus Density

The variation of the cypress and rockrose permittivity versus density was also studied in this work. The volume of biomass for all samples was kept constant while the mass ranged from 1 to 1.8 g. The mass sample was measured by means of a weighing scales from GRAM, model SV, with an accuracy of 0.1 g. The sample volume of 4.17 cm^3^ was obtained considering a cylindrical volume of a 10.3 mm diameter (the test tube specification) and a filling height of 4.8 cm.

The test tubes were filled slowly with biomass driblets while shaking the tube after each load in order to reduce as many air gaps within the sample as possible. The first set of samples had 1 g of mass, and some pressure was applied on them until their filling level reached a mark on the test tube, matching the required initial density of 0.24 g/cm^3^. Higher densities were sequentially achieved by increments of 0.2 g and compacting the biomass until reaching the same height level as before, so its volume was kept constant.

The studied values of density for both cypress and rockrose biomass are shown in Table 1.

The minimum density obtained for both cypress and rockrose samples was 0.24 g/cm^3^, whereas the maximum density was 0.43 g/cm^3^. The permittivity versus density results were computed as the average of three samples per density value and three measurements per sample.

During density-dependent measurements, the sample temperature was fixed to 28 °C (environment temperature). The dry-basis moisture content was 100% for both cypress and rockrose during all measurements.

### 2.5. Measurement of the Influence of Moisture Content over the Biomass Permittivity

Another study carried out in this work consisted of measuring the dielectric properties versus the moisture content of biomass samples. In order to reduce the moisture content, three biomass samples of both cypress and rockrose with densities of 0.43 gr/cm^3^ were introduced in test tubes and then they were gradually dried.

The process of drying was accomplished as follows: All biomass samples were introduced in a traditional electrical oven during one hour at a fixed temperature of 105 °C. After each drying period, we extracted the samples and waited until they reached the room temperature of 28 °C. Then, the mass of each sample was weighted in order to know the amount of evaporated water and the dielectric properties were measured in the DKV. This procedure was repeated until all test tubes were completely dried, i.e., the mass of each test tube was the same after two consecutive drying periods in the traditional oven. The dry mass of each sample was then obtained as the last sample mass measured in this procedure.

In this work, the moisture content, *X*, was expressed as a dry basis as shown in Equation (1):

(1) $$X\left(\%\right)=100\cdot \frac{m_{w}-m_{d}}{m_{d}}$$

where *m_w_* (gr) is the sample mass with some moisture inside and *m_d_* (gr) is the mass of the dried sample. Please be aware that $m_{w}-m_{d}$ represents the mass of the water content, and therefore *X* accounts for the mass water versus the dried solid mass.

The results of the permittivity values as a function of the moisture content were computed using the averaged values from three samples of each biomass type (cypress and rockrose) with a density of 0.43 g/cm^3^.

## 3. Results

### 3.1. Cypress Biomass Permittivity Measurements

The obtained measurements for cypress biomass permittivity are shown in this subsection. The permittivity dependence on temperature, density, and moisture content for cypress biomass samples are shown and discussed in the next sections. Blue circles (•) are used in the figures for representing the average measured data of the dielectric constant and red triangles (**‣**) for the loss factor average measurements.

#### 3.1.1. Permittivity Dependence on Temperature

**Figure 3:**
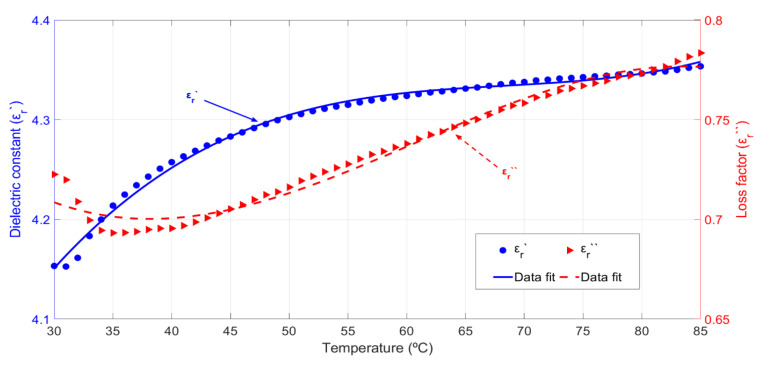
Cypress biomass permittivity behavior versus temperature and polynomial data fit for an average sample density equal to 0.34 g/cm^3^ and an average dry-basis moisture content equal to 100%.

Figure 3 shows the average behavior of the dielectric constant and loss factor of cypress biomass samples versus temperature in the range 30–85 °C. The density of all cypress samples was 0.34 g/cm^3^ and the average dry-basis moisture content was around 100%. As it can be observed from the obtained results, the dielectric constant increases with increasing sample temperatures as well as the loss factor. The increment of the dielectric constant is around 4.8% and the increment of the loss factor is around 8.8% for the whole temperature range. This implies an average relative increase of 0.09% per °C for the dielectric constant and 0.16% per °C for the loss factor. Thus, temperature affects the material losses more in this case.

The cypress biomass permittivity dependence on temperature was modelled by fitting the polynomial expressions to the experimental data, and in this way, we obtained Equations (2) and (3) for the dielectric constant and loss factor temperature dependence, respectively:

(2) $$\varepsilon_{r}^{\prime }\left(T\right)=3.4+0.04\cdot T-0.0006\cdot T^{2}+2.8\cdot 10^{-6}\cdot T^{3}$$

(3) $$\varepsilon_{r}^{\prime \prime }\left(T\right)=0.96-0.016\cdot T+0.0003\cdot T^{2}-1.65\cdot 10^{-6}\cdot T^{3}$$

where *T* is the biomass sample temperature expressed in °C. The root mean square error (RMSE) for the dielectric constant and loss factor fit is, respectively, 0.004 and 0.0043.

#### 3.1.2. Permittivity Dependence on Sample Density

**Figure 4:**
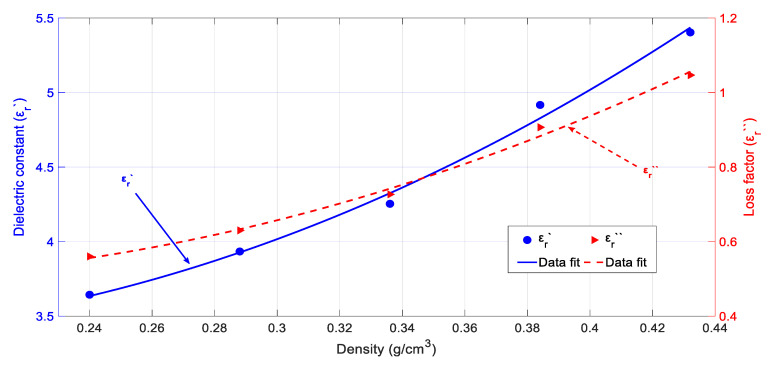
Dielectric properties for cypress depending on the sample density and polynomial fit at room temperature (28 °C) and an average moisture content equal to 100%.

Figure 4 shows the behavior of the cypress biomass permittivity versus density. As it can be observed, the higher the density, the higher the values of both the dielectric constant and loss factor, as expected. High variations with density were detected for both the dielectric constant and loss factor (i.e., a variation above 48% for the dielectric constant and a variation above 91% for the loss factor), with its influence being much more important than temperature. Therefore, the absorption of microwave energy can be modulated by pressing or not the biomass product inside the microwave applicators and can be used as a design parameter.

The continuous variation of both the dielectric constant and loss factor versus density was also modelled by fitting two polynomic expressions to the experimental data, thus obtaining Equations (4) and (5), respectively:

(4) $$\varepsilon_{r}^{\prime }\left(\rho \right)=3.75-5.96\cdot \rho +22.83\cdot \rho^{2}$$

(5) $$\varepsilon_{r}^{\prime \prime }\left(\rho \right)=0.65-2.05\cdot \rho +6.92\cdot \rho^{2}$$

where *ρ* is the sample density expressed in g/cm^3^. The RMSE for the dielectric constant and loss factor fit is, respectively, 0.05 and 0.01.

#### 3.1.3. Permittivity Dependence on Dry-Basis Moisture Content

**Figure 5:**
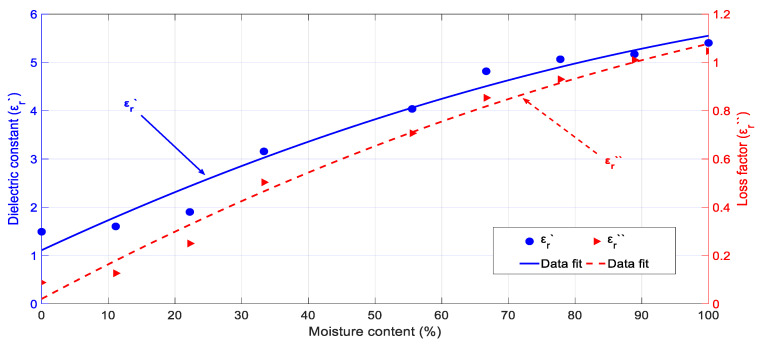
Dielectric properties of cypress biomass depending on the dry-basis moisture content for a 0.43 g/cm^3^ initial density and room temperature (28 °C).

Figure 5 shows the cypress biomass permittivity evolution versus the dry-basis moisture content for samples with an initial density of 0.43 g/cm^3^. All measurements were carried out at 28 °C. From the obtained results, one can conclude that for moisture content values below 70%, both the loss factor and the dielectric constant continuously decrease with decreasing moisture content values, whereas for values above 70%, a stabilization for those magnitudes occurs. The RMSE values obtained for the polynomial fit are higher than in the previous measurements and this might be explained because of the heterogeneous composition of biomass samples, which might cause different drying rates in their components. The fact that drying shrinks the stems, leaves, and sprouts at different velocities can also be an additional explanation for this higher data dispersion.

An important conclusion for microwave-processing protocols is that higher absorption rates and efficiencies will be obtained when the cypress biomass has higher moisture content levels and drying processes due to storage have not occurred yet.

Quadratic polynomials were used to interpolate the experimental data of both the dielectric constant and loss factor of cypress biomass versus the dry-basis moisture content. Equations (6) and (7) show the obtained polynomials for both magnitudes:

(6) $$\varepsilon_{r}^{\prime }\left(X\right)=1.11+6.4\cdot X-1.96\cdot X^{2}$$

(7) $$\varepsilon_{r}^{\prime \prime }\left(X\right)=0.02+1.48\cdot X-0.42\cdot X^{2}$$

where *X* is the moisture content in dry basis. The RMSE values for the dielectric constant and loss factor fitting are, respectively, 0.27 and 0.04.

### 3.2. Rockrose Biomass Permittivity Measurements

The obtained results for rockrose biomass permittivity are shown in this subsection versus temperature, density, and moisture content variations. The results are discussed and compared to the cypress biomass data.

#### 3.2.1. Permittivity Dependence on Temperature

**Figure 6:**
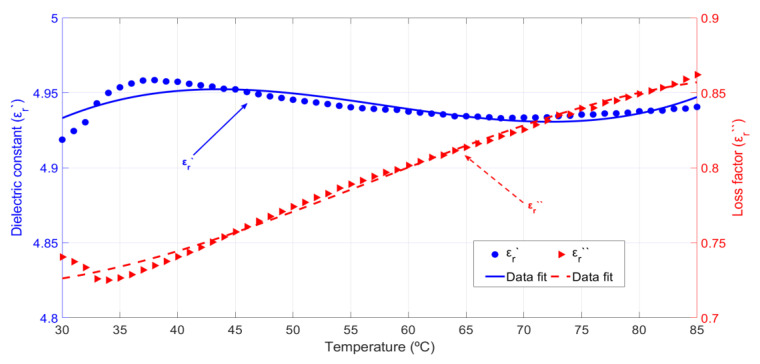
Rockrose biomass permittivity behavior versus temperature and polynomial data fit. The density of samples equal to 0.3 g/cm^3^ and the moisture content (dry basis) is around 100%.

Figure 6 shows the variation of the permittivity versus the temperature for the rockrose biomass samples from 30 to 85 °C. The dielectric constant hardly varies for the whole range of temperatures. The loss factor, however, shows a clear increasing trend, as happened with the cypress biomass measurements. Its values range from 0.74 to 0.86, which implies a 16% increment for the whole temperature range and an average relative increase of 0.29% per °C. The density of the samples was 0.3 g/cm^3^ and the moisture content (dry basis) was around 100% during these measurements.

Equations (8) and (9) show the polynomial fit to the permittivity data versus temperature (*T,* °C) for the rockrose biomass samples:

(8) $$\varepsilon_{r}^{\prime }\left(T\right)=4.65+0.02\cdot T-0.0003\cdot T^{2}+1.8\cdot 10^{-6}\cdot T^{3}$$

(9) $$\varepsilon_{r}^{\prime \prime }\left(T\right)=0.77-0.0047\cdot T+0.0001\cdot T^{2}-8\cdot 10^{-7}\cdot T^{3}$$

where the RMSE values for the dielectric constant and loss factor are, respectively, 0.0048 and 0.0039.

#### 3.2.2. Permittivity Dependence on Sample Density

**Figure 7:**
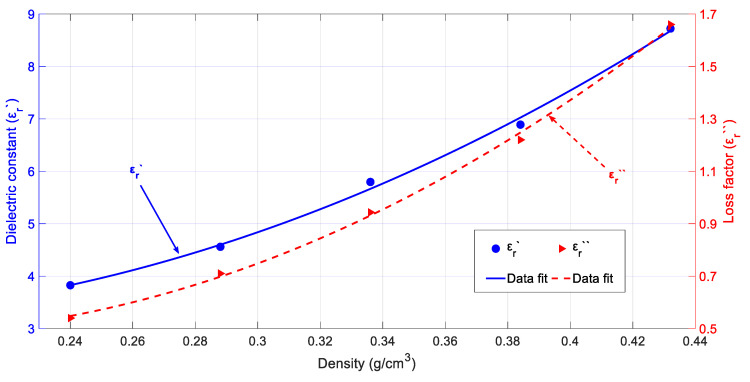
Dielectric properties’ measurements and polynomial data fit for rockrose depending on the sample density. Carried out at 28 °C and a moisture content (dry basis) of around 100%.

Figure 7 shows the rockrose biomass permittivity dependence on density. The measurements were carried out at room temperature and for a moisture content equal to 100%. One can conclude from the obtained results that the bigger the density, the higher the values for both the dielectric constant and loss factor. This behavior is very similar to the one observed for the cypress biomass samples.

Very important permittivity variations with density were detected as in the case of cypress biomass (i.e., a variation of around 125% for the dielectric constant and 200% for the loss factor), with its influence being much more important than temperature. The behavior is very similar to that of cypress biomass, although, in this case, the dielectric constant and loss factor values are larger than those for cypress.

Equations (10) and (11) show, respectively, the polynomial fit for the rockrose biomass dielectric constant and loss factor as a function of the sample density:

(10) $$\varepsilon_{r}^{\prime }\left(\rho \right)=4.38-17.59\cdot \rho +63.74\cdot \rho^{2}$$

(11) $$\varepsilon_{r}^{\prime \prime }\left(\rho \right)=1.05-6.42\cdot \rho +18.08\cdot \rho^{2}$$

where the RMSE value for the fitting of the dielectric constant is 0.09 and the loss factor one is 0.02.

#### 3.2.3. Permittivity Dependence on Dry-Basis Moisture Content

**Figure 8:**
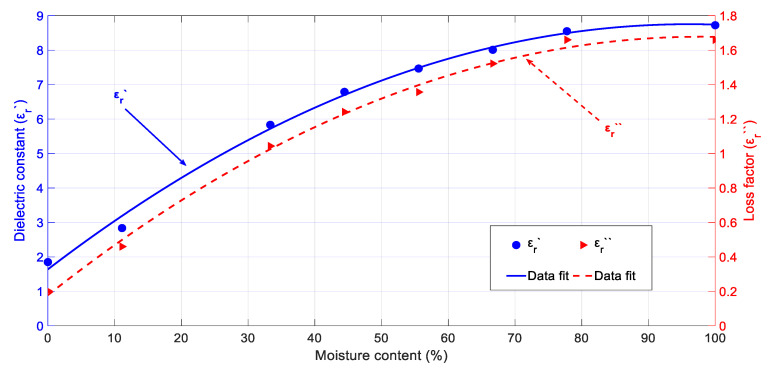
Dielectric properties for rockrose depending on the moisture content (dry basis) for a 0.43 g/cm^3^ initial density and room temperature (24 °C).

Figure 8 shows the rockrose permittivity dependence on the dry basis moisture content at room temperature. The initial density of all samples was around 0.43 g/cm^3^. One can conclude from the results that both the dielectric constant and loss factor increase their values with increasing moisture content levels. Data dispersion was also observed, although in a lower degree (lower RMSE values) than in the case of cypress. A very important variation of the dielectric constant and loss factor was found when varying the dry basis moisture content from 100% to 0%. The dielectric constant varied in a range from 8.7 to 1.8 whereas the loss factor varied from 1.65 to 0.2.

The measured rockrose biomass permittivity values were close to those ones obtained from the cypress biomass samples. Again, for microwave-assisted extraction purposes, it seems more convenient for efficient processing to irradiate the biomass samples with a higher moisture content since microwave absorption will be higher. However, this can be a setback when driving big payload volumes, so the penetration depth can be managed by handling biomass samples with a lower moisture content.

Equations (12) and (13) show, respectively, the polynomial fit of the dielectric constant and loss factor data of rockrose biomass samples as a function of the moisture content:

(12) $$\varepsilon_{r}^{\prime }\left(X\right)=1.64+14.81\cdot X-7.71\cdot X^{2}$$

(13) $$\varepsilon_{r}^{\prime \prime }\left(X\right)=0.18+3.1\cdot X-1.56\cdot X^{2}$$

where *X* is the dry-basis moisture content of the rockrose samples. The RMSE values for the dielectric constant and loss factor fitting are 0.16 and 0.03, respectively.

## 4. Discussion

Permittivity measurements for biomass samples of cypress and rockrose were presented in this work. They were carried out with resonant techniques near the 2.45 GHz ISM frequency. The dependence of both the dielectric constant and loss factor versus temperature, density, and moisture content was shown.

The obtained results for both rockrose and cypress biomass samples indicate that temperature has a limited influence on the dielectric constant, at least for temperatures ranging from 30 to 85 °C. However, the loss factor of both cypress and rockrose biomass increases around 8.8% and 16%, respectively, in that temperature range with increasing temperatures.

It must be remarked that this behavior is different from that measured for pure water as shown in [28]. Whereas pure water shows decreasing values for its dielectric constant and loss factor when temperature increases, the cypress biomass dielectric constant increases and the real part of rockrose biomass permittivity stays constant. Additionally, the loss factors of both kinds of biomass increase with rising temperatures.

The explanation for this behavior can be found in [29,30]: When temperature increases, free water is released from its bound state inside the biomass cells and, therefore, the dielectric constant and, specially, the loss factor increase their values. Therefore, the increased mobility of water within plants due to higher temperatures compensates for the effect that temperature has on pure water.

Density has a much more important influence on both the dielectric constant and loss factor values. Dielectric constant variations range from 48% to 125% for cypress and rockrose, respectively, whereas the loss factor variations versus density range from 91% to 200% for cypress and rockrose, respectively. Therefore, density should be carefully considered when designing an MAE applicator since it can largely affect its efficiency and temperature distribution.

High density values for cypress or rockrose biomass will ensure high loss factor values and, therefore, a high capacity for microwave absorption. This usually leads to high power efficiency values of the microwave applicator and high heating rates. However, the penetration depth will decrease with increasing the density values and therefore uniform heating will be more difficult as the density grows since most microwave energy will dissipate near the biomass surface. Therefore, a compromise between microwave absorption and uniform heating patterns should be considered when choosing the biomass density.

Moisture content also has a remarkable impact on the permittivity values. At least, two important conclusions can be obtained from the results: The first one is that higher moisture contents provide higher microwave absorption rates since the loss factor grows with increasing moisture content values. However, high moisture contents will lead to low penetration depth values and this will hinder uniform heating in the biomass volume. Therefore, there must be a compromise between microwave absorption and uniformity similar to that described for the biomass density of cypress and rockrose.

Additionally, some dispersion of the experimental data was observed when the moisture content changed and this might be explained due to the fact that the drying rates for stems, leaves, or tree branches are different and the composition of biomass is very heterogeneous.

Although the conclusions for biomass permittivity evolution versus temperature, density, and moisture content should be considered valid only around the 2.45 GHz ISM frequency, most of them will also reproduce at lower and higher microwave frequencies, where dipolar rotation is the driving mechanism for dielectric losses.

It is more complex is to consider the combined effect of different parameters. For instance, when samples lose moisture, it is expected that the sample density will also change with respect to the initial value that has been taken in each set of measurements. Therefore, the real variation of the permittivity determined by the combined effect of both parameters should be assumed to be different than that shown for each individual parameter. This is so because the variation in moisture for the cypress or rockrose is not independent of its mass density. A mutual relationship between temperature, moisture, and density determines the expected values for permittivity and future works are envisaged in that direction.

The estimation of the actual distribution of permittivity of the cypress or rockrose biomass within the microwave cavity in MAE processes is a complex task since the biomass will be pressed by blades and other transport elements and thus its density will vary evenly along the microwave applicator. Additionally, the moisture content and temperature distributions will vary during the microwave irradiation process.

However, for microwave applicator designing processes knowing the limits of biomass permittivity is often enough to provide good power efficiency levels, above 90%, and uniform temperature profiles. In this case, designers can test the best and worst cases and the response of the applicator both in terms of power reflection and the expected heating patterns

Finally, the obtained values of permittivity for both rockrose and cypress biomass show that these materials can be irradiated with microwave energy in order to extract profitable chemical substances. The irradiation would be more efficient for higher values of moisture content and density, although a compromise between the absorption and power efficiency should be taken into account when designing MAE applicators.

### Implications for Sensor Design

The permittivity measurements carried out in this work for cypress and rockrose biomass will be useful for sensor design since the correlations of biomass permittivity with temperature, moisture content, and density are very clear.

The loss factor increases versus temperature with a biunivocal relationship in the range 35–85 °C for both cypress and rockrose biomass samples. This indicates that it would be possible to estimate the plant temperature increase due to undesired fire spots, ray strokes, or problems in greenhouses by using directive radio links since a temperature increment would lead to an important attenuation rise and a decrease in the received power.

This temperature-driven loss factor increment is directly correlated with the conversion of bound water into free water and therefore it could be related to the dehydration of the plants and their health. Thus, transmission-based microwave sensors could also measure the temperature stress resistance of cypress and rockrose plants, even for different varieties of the same species.

There is also a biunivocal relationship between the density and permittivity. In fact, both the dielectric constant and loss factor continuously increase as the density rises. Since density affects both the microwave power absorption capacity and penetration depth, it would be important to control this parameter when feeding microwave applicators. In this case, several possibilities can be implemented, such as resonant sensors or devices based on the reflection and/or transmission of microwave energy.

A sensor indicating the moisture content of biomass would also be useful in order to introduce the biomass into the microwave applicator at the optimal state for microwave-assisted extraction in terms of the penetration depth and microwave energy absorption. Obviously, controlling the moisture content of biomass would provide repeatability and the possibility to optimize the MAE process. Again, resonant techniques or sensors based on transmission/reflection configurations could be implemented since both the loss factor and the dielectric constant monotonously grow with increasing values of the moisture content.

Another application of density and moisture monitoring would be the estimation of the number of plants per square meter in a forest, which could be carried out with directive antennas. The attenuation of the radio link will be directly linked to the forest density and moisture content of the plants.

Future work is envisaged in this direction in order to correlate permittivity measurements to different biomass parameters and to develop non-invasive microwave sensors.

## Figures and Tables

**Table 1:** Biomass density dependence on mass in the test tubes.

Mass (g)	Density (g/cm^3^)
1	0.24
1.2	0.29
1.4	0.34
1.6	0.38
1.8	0.43
